# Sodium Intake Among Persons Aged ≥2 Years — United States, 2013–2014

**DOI:** 10.15585/mmwr.mm6612a3

**Published:** 2017-03-31

**Authors:** Zerleen S. Quader, Lixia Zhao, Cathleen Gillespie, Mary E. Cogswell, Ana L. Terry, Alanna Moshfegh, Donna Rhodes

**Affiliations:** ^1^Division for Heart Disease and Stroke Prevention, National Center for Chronic Disease Prevention and Health Promotion, CDC; ^2^Division for Health and Nutrition Examination Surveys, National Center for Health Statistics, CDC; ^3^Food Surveys Research Group, Agriculture Research Services, United States Department of Agriculture, Beltsville, Maryland.

High sodium consumption can increase hypertension, a major risk factor for cardiovascular diseases (*1*). Reducing sodium intake can lower blood pressure, and sodium reduction in the U.S. population of 40% over 10 years might save at least 280,000 lives (*2*). Average sodium intake in the United States remains in excess of *Healthy People 2020* objectives,* and monitoring sources of sodium in the U.S. population can help focus sodium reduction measures (*3*,*4*). Data from 2013–2014 What We Eat in America (WWEIA), the dietary intake portion of the National Health and Nutrition Examination Survey (NHANES),^†^ were analyzed to determine the ranked percentage sodium contribution of selected food categories and sources of sodium intake from all reported foods and beverages, both overall and by demographic subgroups. These latest data include updated food codes and separate estimates for intake among non-Hispanic Asians.^§^ In 2013–2014, 70% of dietary sodium consumed by persons in the United States came from 25 food categories; breads were the top contributor, accounting for 6% of sodium consumed. A majority of sodium consumed was from food obtained at stores; however, sodium density (mg/1,000 kcal) was highest in food obtained at restaurants. A variety of commonly consumed foods contributes to U.S. sodium intake, emphasizing the importance of sodium reduction across the food supply (*4*).

NHANES is a nationally representative sample of the U.S. noninstitutionalized population that uses a multistage probability sampling design, with oversampling of certain population subgroups, including non-Hispanic blacks, Hispanics, and since 2011–12, non-Hispanic Asians. In 2013–2014, interviews and examinations were conducted among 9,813 participants (68.5% overall response rate), 8,067 of whom were aged ≥2 years and had a complete and reliable 24-hour dietary recall. The dietary recall was conducted in the NHANES mobile examination center, and information on types and amounts of all foods and beverages consumed during the previous 24 hours were self-reported by the participant to a trained interviewer using U.S. Department of Agriculture’s (USDA’s) automated multiple-pass method.^¶^ Each reported food or beverage was assigned a food code from the USDA Food and Nutrient Database for Dietary Studies (FNDDS) with corresponding nutrient content by weight.** Nutrient intake from each food for each person was estimated by multiplying the reported amount of food consumed by the nutrient intake per amount. The amount of salt added to food at the table was not collected; thus estimates of sodium intake exclude salt added at the table.^††^ Each of the 8,537 FNDDS food codes was assigned to one of 153 WWEIA food categories, grouped on the basis of consumption and nutrient content.^§§^ Similar categories were further combined into 109 categories for this analysis.

The top 25 food categories contributing to sodium consumption were identified and ranked based on their contribution to sodium consumed from all reported foods and beverages, excluding salt added at the table (the sum of the amount of sodium consumed from all foods within a specific category, or source, for all persons, divided by the sum of the amount of sodium consumed from all foods for all persons, and multiplied by 100). Sodium density was used to account for differences in the amount of calories consumed. Analyses were conducted using statistical software that accounts for the complex survey design, and for all estimates, 1-day dietary sample weights were used.

The mean daily sodium intake from foods and beverages among the U.S. population aged ≥2 years was 3,409 mg, and the mean sodium density was 1,683 mg/1,000 kcal. Across age subgroups, sodium intake was highest among persons aged 20–50 years (Table 1). Men had significantly higher sodium intake than did women (T-tests, p<0.001) and non-Hispanic Asians consumed a more sodium-dense diet and fewer calories compared with non-Hispanic whites (T-tests, p<0.05) (Table 2). Overall, 44% of sodium consumed came from 10 food categories, with 70% from 25 food categories, ranging from 3.8% to 6.2% from the top five: breads (rank = 1), pizza (2), sandwiches (3), cold cuts and cured meats (4), and soups (5), to 1.3% from rice (25) (Table 1). For almost all population subgroups, the top five food categories contributing to sodium intake were among the top 10 categories for the overall population aged ≥2 years. Exceptions to this were milk (fourth highest contributor to sodium intake among children aged 2–5 years), meat mixed dishes (fifth highest contributor among adults aged ≥71 years), other Mexican mixed dishes (fifth highest contributor to sodium among Hispanics), and rice and soy-based condiments (second and fifth highest contributors to sodium, respectively, among Asians) (Table 1) (Table 2).

**TABLE 1:** Mean intakes of sodium and energy, mean sodium density, and ranked percentage sodium contribution of selected food categories* among persons aged ≥2 years, by age groups — What We Eat in America (WWEIA), National Health and Nutrition Examination Survey, United States, 2013–2014

Characteristic		Age group (yrs)								
		≥2	2–19	2-5	6–11	12–19	≥20	20–50	51–70	≥71
Sample size		8,067	3,020	677	1,047	1,296	5,047	2,733	1,645	669
Mean sodium intake (mg)**^†^**		3,409	3,033	2,248	2,992	3,411	3,529	3,754	3,343	2,928
Mean energy intake (kcal)**^†^**		2,079	1,885	1,480	1,921	2,038	2,141	2,271	2,042	1,773
Mean sodium density (mg/1,000 kcal)**^†^**		1,683	1,627	1,534	1,574	1,706	1,701	1,703	1,699	1,695
**Rank** ^§^	**WWEIA food category**	**% contribution** ^¶^								
1	Yeast breads¶	6.2	5.7	6.3	5.7	5.5	6.4	5.5	7.6	8.1
2	Pizza	5.9	8.3	5.7	9.8	8.0	5.3	7.1	2.9	1.6
3	All single code sandwiches**	5.7	6.5	4.9	6.4	7.0	5.5	6.4	4.2	4.0
4	Cold cuts and cured meats	5.4	4.1	4.0	3.5	4.4	5.7	5.6	5.6	6.8
5	Soups	3.8	3.1	3.3	3.4	2.9	4.0	3.5	4.4	6.0
6	Burritos and tacos	3.8	4.2	2.6	4.8	4.2	3.7	4.5	2.9	1.1
7	All savory snacks^††^	3.7	5.2	7.9	4.9	4.6	3.3	3.2	3.5	2.6
8	Chicken, whole pieces	3.7	3.0	2.2	2.6	3.4	3.9	4.1	3.7	2.5
9	Cheese^§§^	3.5	3.7	4.1	3.7	3.6	3.4	3.4	3.6	2.8
10	Eggs and omelets	2.6	2.0	2.6	1.9	1.9	2.8	2.8	2.8	3.1
11	Meat mixed dishes	2.5	2.0	1.1	2.3	2.1	2.7	2.1	3.4	4.1
12	Pasta mixed dishes, excludes macaroni and cheese	2.5	2.7	2.9	2.5	2.7	2.4	2.3	2.7	2.4
13	Bacon, frankfurters, and sausages	2.0	2.2	4.5	1.7	1.8	2.0	1.7	2.4	2.0
14	Tomato-based condiments	1.8	1.6	1.5	1.7	1.7	1.9	2.2	1.7	0.9
15	Salad dressings and vegetable oils	1.8	1.4	0.7	1.1	1.8	2.0	1.9	2.0	2.3
16	Other Mexican mixed dishes	1.8	2.4	3.3	2.6	2.0	1.6	2.1	1.1	0.8
17	Poultry mixed dishes	1.7	1.1	0.7	0.9	1.3	1.8	1.6	2.4	1.7
18	All plain milk	1.6	2.9	4.9	2.9	2.3	1.3	1.1	1.3	2.2
19	Fish	1.5	0.6	1.4	0.5	0.5	1.8	1.5	2.3	1.8
20	Mashed potatoes and white potato mixtures	1.5	1.3	0.6	0.7	1.9	1.6	1.3	1.8	2.8
21	All ready-to-eat cereal	1.5	2.4	3.0	2.6	2.2	1.2	1.0	1.3	2.3
22	French fries and other fried white potatoes	1.4	1.3	1.1	1.3	1.4	1.4	1.4	1.5	0.9
23	Other vegetables and combinations	1.4	0.6	0.9	0.5	0.6	1.6	1.3	1.9	2.0
24	Cakes and pies	1.4	1.0	0.9	0.9	1.0	1.5	1.2	1.8	2.1
25	Rice	1.3	1.3	1.5	1.5	1.2	1.3	1.5	1.0	1.1
28^¶¶^	Cookies and brownies	1.2	1.4	2.0	1.7	1.1	1.1	1.0	1.2	1.9
29^¶¶^	Chicken patties, nuggets and tenders	1.2	2.7	2.6	2.9	2.6	0.8	0.9	0.6	0.5
30^¶¶^	Stir-fry and soy-based sauce mixtures	1.1	1.5	0.4	0.7	—^***^	1.0	1.1	0.9	0.7
35^¶¶^	Macaroni and cheese	1.0	1.5	1.5	2.3	1.0	0.8	0.8	0.8	0.8
36^¶¶^	Pancakes, waffles, and French toast	0.9	1.9	1.7	2.6	1.6	0.6	0.6	0.7	0.5
54^¶¶^	All flavored milk	0.4	1.2	1.5	1.8	0.7	0.2	0.3	0.1	0.1
	All other categories	24.2	19.2	17.7	17.6	20.7	25.4	25.0	26.0	27.5

* The percentage (%) sodium consumed is defined as the sum of the amount of sodium consumed from each specific WWEIA food category for all participants in the designated group, divided by the sum of sodium consumed from all food categories for all participants in the designated group, multiplied by 100. All estimates use one 24-hour dietary recall, take into account the complex sampling design, and use the 1-day diet sample weights to account for nonresponse and weekend/weekday recalls.

^†^ All estimates use one 24-hour dietary recall, take into account the complex sampling design, and use the 1-day diet sample weights to account for nonresponse and weekend/weekday recalls.

^§^ Rank based on the percentage of sodium consumed for overall U.S. population aged ≥2 years. Columns of other age groups are ordered by the ranking for the overall U.S. population aged >2 years. WWEIA food categories available at http://www.ars.usda.gov/Services/docs.htm?docid=23429.

^¶^ Yeast breads, rolls, buns, bagels, and English muffins.

** Sandwiches, identified by a single WWEIA food code, include burgers, frankfurter sandwiches, chicken/turkey sandwiches, egg/breakfast sandwiches, and other sandwiches.

^††^ Chips, popcorn, pretzels, snack mixes, and crackers.

^§§^ Natural and processed cheese.

^¶¶^ Food categories that are in the top 20 contributors to sodium within an age subgroup but not in the top 25 overall.

*** Estimates are statistically unreliable, relative standard error >30%.

**TABLE 2:** Mean intakes of sodium and energy, mean sodium density, and ranked percentage sodium contribution of selected food categories* among persons aged ≥2 years, by sex and race/ethnicity — What We Eat In America (WWEIA), National Health and Nutrition Examination Survey, United States, 2013–2014

Characteristic		Sex		Race/Ethnicity			
		Male	Female	Non-Hispanic white	Non-Hispanic black	Hispanic	Non-Hispanic Asian
Sample size		3,934	4,133	3,044	1,762	2,122	789
Mean sodium intake (mg)^†^		3,915	2,920^§^	3,407	3,381	3,424	3,538
Mean energy intake (kcal)^†^		2,382	1,786^§^	2,080	2,133	2,104	1,853^¶^
Mean sodium density (mg/1,000 kcal)^†^		1,681	1,685	1,681	1,636	1,659	1,946^¶^
**Rank** ^**^	**WWEIA food category**	**% contribution**					
1	Yeast breads^††^	6.3	6.2	6.9	5.3	4.7	5.7
2	Pizza	6.2	5.5	5.8	6.8	5.5	3.2
3	All single code sandwiches^§§^	6.0	5.4	5.3	8.3	6.4	2.5
4	Cold cuts and cured meats	6.0	4.5	6.5	3.6	3.6	2.8
5	Soups	3.5	4.3	3.2	2.6	5.1	10.5
6	Burritos and tacos	4.1	3.4	3.1	1.8	8.9	0.6
7	All savory snacks^¶¶^	3.3	4.2	3.9	4.2	2.8	1.9
8	Chicken, whole pieces	4.0	3.2	3.2	5.7	4.0	3.8
9	Cheese***	3.4	3.5	3.9	2.8	2.9	1.5
10	Eggs and omelets	2.7	2.6	2.4	2.7	3.3	2.6
11	Meat mixed dishes	2.7	2.3	2.8	2.9	1.6	2.4
12	Pasta mixed dishes, excludes macaroni and cheese	2.7	2.3	2.7	2.4	1.7	1.9
13	Bacon, frankfurters, and sausages	2.1	1.9	2.1	3.0	1.3	1.2
14	Tomato-based condiments	1.9	1.7	1.9	1.7	2.0	1.0
15	Salad dressings and vegetable oils	1.5	2.3	2.0	1.8	1.5	1.0
16	Other Mexican mixed dishes	1.7	1.9	1.3	0.5	4.8	0.4
17	Poultry mixed dishes	1.6	1.7	1.9	0.8	1.3	2.0
18	All plain milk	1.8	1.5	1.8	1.0	1.6	1.4
19	Fish	1.5	1.5	1.4	2.6	1.0	2.2
20	Mashed potatoes and white potato mixtures	1.6	1.5	1.9	1.4	0.9	0.5
21	All ready-to-eat cereal	1.5	1.5	1.5	1.4	1.5	0.9
22	French fries and other fried white potatoes	1.4	1.3	1.4	1.6	1.2	1.0
23	Other vegetables and combinations	1.2	1.6	1.5	1.0	0.8	3.0
24	Cakes and pies	1.1	1.7	1.3	1.8	1.2	1.2
25	Rice	1.2	1.4	0.6	1.6	1.6	7.9
26^†††^	Beef, excludes ground	1.3	1.1	1.2	1.1	1.6	1.0
27^†††^	Rice mixed dishes	1.1	1.3	0.9	1.4	2.1	1.2
29^†††^	Chicken patties, nuggets, and tenders	1.1	1.3	1.1	1.7	1.2	—^§§§^
30^†††^	Stir-fry and soy-based sauce mixtures	1.1	1.1	1.1	—^§§§^	1.2	2.7
33^†††^	Biscuits, muffins, and quick breads	0.9	1.0	1.0	1.7	0.5	—^§§§^
37^†††^	Beans, peas, and legumes	0.9	0.9	0.6	1.0	2.1	0.9
41^†††^	Fried rice and lo/chow mein	0.7	0.8	0.7	0.7	0.5	2.4
43^†††^	Soy-based condiments	—^§§§^	0.6	—^§§§^	—^§§§^	0.5	3.4
46^†††^	Tortillas	0.6	0.5	0.3	0.0	1.9	0.4
55^†††^	Egg rolls, dumplings, and sushi	0.4	0.4	0.4	0.1	—^§§§^	1.5
	All other categories	20.3	22.1	21.9	22.1	16.9	21.9

* The percentage (%) sodium consumed is defined as the sum of the amount of sodium consumed from each specific WWEIA food category for all participants in the designated group, divided by the sum of sodium consumed from all food categories for all participants in the designated group multiplied by 100. All estimates use one 24-hour dietary recall, take into account the complex sampling design, and use the 1-day diet sample weights to account for nonresponse and weekend/weekday recalls.

^†^ All estimates use one 24-hour dietary recall, take into account the complex sampling design, and use the 1-day diet sample weights to account for nonresponse and weekend/weekday recalls.

^§^ Statistically significant difference (p<0.001) in mean sodium and energy intakes compared with males.

^¶^ Statistically significant difference (p<0.001) compared with non-Hispanic whites.

^**^ Rank based on percentage of sodium consumed for overall U.S. population aged ≥2 years. Columns of other sex and race/ethnic groups are ordered by this ranking. WWEIA food categories available at http://www.ars.usda.gov/Services/docs.htm?docid=23429.

^††^ Yeast breads, rolls, buns, bagels, and English muffins.

^§§^ Sandwiches identified by a single WWEIA food code, includes burgers, frankfurter sandwiches, chicken/turkey sandwiches, egg/ breakfast sandwiches, and other sandwiches.

^¶¶^ Chips, popcorn, pretzels, snack mixes, and crackers.

*** Natural and processed cheese.

^†††^ Food categories that are in the top 20 contributors to sodium within an age subgroup but not in the top 25 overall.

^§§§^ Estimates are statistically unreliable, relative standard error >30%.

The majority of sodium consumed came from food obtained at stores (60.8%), followed by fast food/pizza restaurants (16.7%), restaurants with waitstaff (10.7%), and school cafeteria or child/adult care center (2% overall; 8.8% among children aged 2–19 years). The remaining sodium (9.8%) was consumed from other listed sources. Among the total population and among children, food obtained from restaurants with waitstaff were the most sodium-dense, whereas among adults, food obtained from cafeterias/care centers were as sodium-dense as those from restaurants (Table 3).

**TABLE 3:** Percentage of sodium consumed* and mean sodium density,^†^ among persons aged ≥2 years, by food source category and age group — What We Eat in America (WWEIA), National Health and Nutrition Examination Survey, United States, 2013–2014

Population groups	Food source category^§^				
	Store	Restaurant with fast food/pizza	Restaurant with waiter/waitress	Cafeteria at school/ child/adult care center	Other
**Total**					
% Contribution (SE)	60.8 (0.6)	16.7 (0.5)	10.7 (0.6)	2.0 (0.1)	9.8 (0.6)
Sodium density (mg/1,000 kcal) (SE)	1,557 (25.6)	1,855 (29.1)	2,119 (32.6)	1,676 (38.0)	1,962 (71.0)
**Children, aged 2–19 years**					
% Contribution (SE)	60.9 (0.6)	16.7 (0.9)	5.4 (0.7)	8.8 (0.7)	8.1 (0.6)
Sodium density (mg/1,000 kcal) (SE)	1,527 (21.5)	1,801 (35.5)	1,972 (59.7)	1,646 (34.6)	1,543 (104.1)
**Adults, aged ≥20 years**					
% Contribution (SE)	60.7 (0.8)	16.7 (0.5)	12.1 (0.7)	*—^¶^*	10.2 (0.6)
Sodium density (mg/1,000 kcal) (SE)	1,566 (34.6)	1,871 (32.2)	2,143 (38.0)	2,179 (366.5)	2,074 (82.7)

**Abbreviation**: SE = standard error.

* The percentage (%) of sodium consumed is defined as the sum of the amount of sodium consumed from each specific food source category for all participants in the designated group, divided by the sum of sodium consumed from all food source categories for all participants in the designated group multiplied by 100. All estimates use one 24-hour dietary recall, take into account the complex sampling design, and use the 1-day diet sample weights to account for nonresponse and weekend/weekday recalls. Standard errors of the estimates are in parentheses.

^†^ A measure that accounts for differences in the amount of calories consumed from foods obtained from each source, defined as mg of sodium per 1,000 kcal.

^§^ Food source categories were analyzed from responses to the question, “Where did you get this (most of the ingredients for this) [food name]?” “Cafeteria at school” and “child care center” were combined in one category. Sources other than those shown were combined under “other” and included “from someone else/gift”, and 19 other sources (e.g., vending machine), including “missing,” “do not know,” and “other/specify”.

^¶^ Estimates are statistically unreliable, relative standard error >30%.

## Discussion

This analysis found that approximately 70% of sodium consumed by the U.S. population aged ≥2 years came from 25 food categories, with 44% from the top 10 categories alone, and provides the most current data on sources of U.S. sodium intake. These results are consistent with previous reports that found that store-bought and restaurant foods are the main contributors to sodium in the diets of persons in the United States, emphasizing the importance of monitoring sodium content of these foods (*5*,*6*). Average U.S. daily sodium intake continues to exceed the *Healthy People 2020* objective of 2,300 mg (*3*).

Since 2007–2008, a majority of the major food categories contributing to population sodium intake have not changed, with the exception of burritos and tacos, which were previously not ranked in the top 10, but were the sixth highest contributor in 2013–2014 (*5*). This might be attributable in part to an actual increase in consumption of these foods, but more likely represents a difference in collection and coding methodology that capture Mexican mixed dishes as a single food code.^¶¶^

In addition, rankings for some food categories differed among racial/ethnic groups. Among Hispanics, burritos and tacos contributed 8.9% of sodium intake, compared with 3.8% among the general population. Most notably, among non-Hispanic Asians, the top two sources of sodium were soups and rice, contributing much more dietary sodium than among other racial/ethnic groups. However, FNDDS food codes for rice include sodium from salt added in cooking; this might partially explain the high contribution to sodium, since rice without salt added is naturally low in sodium. Asian-Americans are the fastest growing racial/ethnic group in the United States; as a group, their diets and cardiovascular health and risk factors might differ from those of other racial/ethnic populations in the United States, although data on this subject are limited (*7*,*8*). The racial/ethnic differences in food types contributing to sodium intake suggest the importance of sodium reduction across the food supply, rather than in just a few categories, to reflect diversity in food choices.

While the majority of sodium was obtained from food purchased at stores, 27% of sodium consumed came from food obtained at restaurants, and restaurant food contributed more sodium per calorie than did food obtained from stores, which supports the need to monitor and reduce sodium levels in food across these venues (*4*,*5*).

The findings in this report are subject to at least five limitations. First, dietary data are self-reported and therefore subject to recall bias and underreporting; however, USDA’s automated multiple-pass method is a valid measure of population level sodium intake (*9*). Second, the results of this analysis are not generalizable to institutionalized populations. Third, the ranking of food categories by their contribution to sodium intake is influenced by methods of categorizing and defining specific foods. For example, sandwiches, when defined as both single-code sandwiches and combinations of individual sandwich ingredients, contribute about one fifth of total sodium intake by U.S. adults (*10*). Fourth, the data are subject to errors in food coding and composition. Finally, estimates of sodium consumption exclude sodium from salt added at the table, which accounts for an estimated 5%–6% of total sodium intake.

Monitoring population sodium intake and sources of sodium can inform measures to reduce sodium content of the food supply. Since publication of previous reports on sodium intake in the U.S. population, initiatives including CDC’s Sodium Reduction in Communities Program,*** New York City’s National Sodium Reduction Initiative,^†††^ and the Healthy Hunger-Free Kids Act^§§§^ are aimed at reducing the sodium content of foods in specific venues and communities, including stores, restaurants and school cafeterias. In 2016, the Food and Drug Administration issued draft voluntary sodium targets to encourage food manufacturers and restaurants to gradually lower the sodium content of food products.^¶¶¶^ Persons can compare Nutrition Facts labels when shopping, choose lower sodium options, and request nutrition information when dining out. The current data can serve as a baseline to monitor changes in the population’s sodium intake and food types contributing to sodium intake overall, and by subgroup to help target initiatives. The results of this study indicate that U.S. sodium intake continues to exceed *Healthy People 2020* targets and comes from a variety of foods and sources. Moderate sodium reduction in the food supply is a key recommended public health strategy to prevent cardiovascular disease (*4*).

**Summary**

**What is already known about this topic?**

Reducing sodium intake can reduce blood pressure; hypertension is a risk factor for cardiovascular disease. According to data from the National Health and Nutrition Examination Survey 2007–2008, average daily U.S. sodium intake was 3,266 mg, exceeding *Healthy People 2020* objectives, and 44% of sodium consumed came from just 10 food types.

**What is added by this report?**

The most recent data, from 2013–2014, indicate that average daily U.S. sodium intake is 3,409 mg (excluding salt added at the table), with 44% of intake from 10 food types and 70% from 25 food types, 61% from food obtained at stores, and highest sodium density (mg/1,000 kcal) from food obtained at restaurants. Food types contributing to intake differ by racial /ethnic group, with current data indicating that non-Hispanic Asians might consume a slightly more sodium-dense diet than that of non-Hispanic whites.

**What are the implications for public health practice?**

Sodium intake remains high and comes from a variety of food types and places. Monitoring differences in types and sources of intake can help focus sodium reduction measures to reduce blood pressure and cardiovascular disease.

## Footnotes

*
https://www.healthypeople.gov/2020/topics-objectives .

†
https://www.cdc.gov/nchs/nhanes/index.htm .

§
https://wwwn.cdc.gov/nchs/nhanes/search/nhanes11_12.aspx .

¶
https://www.ars.usda.gov/News/docs.htm?docid=7710 .

**
https://www.ars.usda.gov/News/docs.htm?docid=12089 .

††
https://www.ars.usda.gov/ARSUserFiles/80400530/pdf/0910/discontinuation%20of%20data%20processig%20step-salt%20adjustment.pdf .

§§
https://www.ars.usda.gov/Services/docs.htm?docid=23429 .

¶¶
https://www.ars.usda.gov/Services/docs.htm?docid=12068 .

*** https://www.cdc.gov/dhdsp/programs/sodium_reduction.htm.

†††
https://www1.nyc.gov/site/doh/health/health-topics/national-salt-reduction-initiative.page .

§§§
https://www.fns.usda.gov/school-meals/healthy-hunger-free-kids-act .

¶¶¶
https://www.fda.gov/Food/IngredientsPackagingLabeling/FoodAdditivesIngredients/ucm253316.htm .
